# Correlation of sino-nasal outcome test and nasal polyp score in dupilumab-treated chronic rhinosinusitis with nasal polyps

**DOI:** 10.1007/s00405-024-08973-7

**Published:** 2024-09-21

**Authors:** Tina Mauthe, Fabio S. Ryser, Catrin Brühlmann, Ayla Yalamanoglu, Christian Meerwein, Urs C. Steiner, Michael B. Soyka

**Affiliations:** 1 Department of Otorhinolaryngology, Head and Neck Surgery, University Hospital Zurich, University of Zurich, Zurich, Switzerland; 2 Faculty of Medicine, University of Zurich, Zurich, Switzerland; 3 Department of Rheumatology and Immunology, University Hospital Bern, University of Bern, Bern, Switzerland; 4 Graduate School for Health Sciences, University of Bern, Bern, Switzerland; 5 Department of Immunology, University Hospital Zurich, University of Zurich, Zurich, Switzerland; 6 Institute of Clinical Chemistry, Inselspital Bern University Hospital, University of Bern, Bern, Switzerland

**Keywords:** Dupilumab, Chronic rhinosinusitis with nasal polyps, Patient related outcome measures, Nasal polyp score, SNOT, Sinonasal outcome test

## Abstract

**Background:**

The alignment between objective scores and patient-reported outcome measures (PROMs) is underexplored. This study aimed to assess changes in Nasal Polyp Score (NPS) and Sino-Nasal Outcome Test (SNOT) scores in chronic rhinosinusitis with nasal polyps (CRSwNP) patients undergoing dupilumab treatment and explore correlations between these scores.

**Methods:**

CRSwNP patients received dupilumab therapy for six months. SNOT-20 German Adapted Version (GAV)/SNOT-22 scores were assessed weekly, and NPS was measured at baseline and after one, three, and six months. Correlations were analyzed using Spearman’s rank correlation and regression analysis.

**Results:**

69 patients were included. After one, three and six months of dupilumab therapy, SNOT and NPS scores improved significantly. Correlation analysis of SNOT and NPS showed significant correlations only within the nasal subscores, along with a weak trend for SNOT-20. Absolute changes over time lacked significance. However, correlation analysis revealed significant associations between relative changes in SNOT score and NPS, irrespective of timing, and when stratified by baseline NPS of 8, 6, and 4 (*r* = -0.54, *p* = 0.01; *r* = -0.44, *p* < 0.001; *r* = -0.7, *p* < 0.001). This was supported by linear regression modeling, suggesting potential predictive capability of NPS reduction on relative SNOT score improvement.

**Conclusion:**

Dupilumab therapy significantly improved subjective and objective CRSwNP scores, exhibiting weak correlations in absolute values for nasal subscores. Furthermore, evidence indicated a correlation between relative changes in SNOT score and NPS, substantiated by predictive capability. This might be due to subjective perception variability, highlighting the suitability of relative change correlation analysis.

## Background

Chronic rhinosinusitis with nasal polyps (CRSwNP) is a prevalent and heterogeneous inflammatory disease, often associated with a significant subjective burden for patients [1, 2]. Patients experience nasal congestion, olfactory loss and rhinorrhea resulting in a reduced quality of life [3, 4].

Advances in understanding the underlying pathophysiology of CRSwNP have led to the increasing importance of immunomodulatory therapies with biologics for CRSwNP patients [5–8]. Notably, the approval of dupilumab (anti-interleukin-4 receptor alpha monoclonal antibody) by the US Food and Drug Administration and the European Medicines Agency in 2019 has revolutionized treatment options for CRSwNP patients [9]. Studies have shown that therapy not only improves nasal-specific symptoms but also other health-related aspects such as decreased nocturnal awakenings, increased concentration, and less frustration [10, 11].

Various assessment tools are accessible for quantifying therapy responses.

The endoscopically determined nasal polyp score (NPS), often referred to as the total polyp score (TPS), is widely used in clinical trials [12–14]. The addition of a PROM to objective endoscopic evaluation methods is strongly recommended to assess treatment response comprehensively [15]. Patient-Related Outcome Measures (PROMs) have the advantage of capturing individual distress and subjective symptomatology, which are crucial criteria for health insurance approval, given the high cost of dupilumab therapy [16, 17]. One of the most qualitatively representative tools that can be utilized is the Sino-Nasal Outcome Test (SNOT), a well-established questionnaire [18].

The extent to which different nasal polyp scoring systems align with PROMs has not been thoroughly explored in existing literature. The European position paper on rhinosinusitis and nasal polyps 2020 emphasizes the lack of literature investigating the correlation between patient-reported and objective outcome measures for CRS [9]. As nasal polyp grading gaining importance as a primary outcome in pharmacological studies and for health insurance approval, several researchers have examined the relationship between objective measures and patient-reported outcome measures [15, 19]. With the predominant conclusion being that the predictive ability of current scoring systems for PROMs is poor and not significant. This underscores the need for further comprehensive research on this topic.

The aim of this study was to evaluate the changes in NPS and SNOT scores in patients with CRSwNP undergoing dupilumab therapy and to investigate the potential correlation between NPS and SNOT scores.

## Methods

Data from two cohorts, the retrospective monocentric cohort study DUPIPOLYP (*n* = 43) and the prospective observational cohort study IMMUNOPOLYP (*n* = 26), collected between June 2020 and November 2022, were analyzed. All participants had signed the written general informed consent form of the University Hospital Zurich (Switzerland) or the study-specific consent form for the IMMUNOPOLYP study. These clinical studies were both conducted with approval from the cantonal ethics committee (BASEC-No. 2021 − 01213 and 2020–02955).

Included in this study were adult patients (≥ 18 years), diagnosed with refractory CRSwNP according to EPOS 2020 [9]. These patients either had a history of previous surgery or were not eligible for surgery. Data from patients who prematurely terminated the study were also included in the analyses.

All patients received Dupixent^®^ (Sanofi) injections subcutaneously every two weeks (300 mg/2 ml dupilumab), alongside nasal saline rinsing and topical steroids. Baseline NPS severity was assessed at first injection, with further consultations at one, three, and six months. For DUPIPOLYP patients additional examinations were conducted one week post-study commencement.

Nasal polyp evaluation involved meticulous video-recorded assessments, with NPS graded based on extension (assessed by summing scores from the right and left nostrils, on a scale of 0 to 8; higher scores indicating a more severe condition) [20].

Patients were instructed to complete the SNOT on a smartphone or computer weekly (SNOT-20 German Adapted Version (GAV) *n* = 43, SNOT-22 *n* = 26), and the data were directly transferred to our clinic database (ENT Statistics, Innoforce, Switzerland). For the analyses, SNOT scores completed within ± 7 days of the consultation were evaluated. The SNOT-20 GAV is a validated instrument for assessing symptoms of colds and upper respiratory diseases, consisting of 20 questions divided into three categories: “primary nasal symptoms” (PNS: 5 questions), “secondary rhinogenic symptoms” (SRS: 6 questions), and “general quality of life” (ALQ: 9 questions) [21, 22].

Symptomatology is classified as mild at 0–10 points, moderate at 11–40 points, moderately severe at 41–69 points, and severe at 51 points or more [23]. As a newly validated German version of the SNOT-22 was available during the study, we followed the international standard with 22 questions. The SNOT-22 test comprises 22 questions divided into five categories: “nasal symptoms,” “sleep,” “ear pain,” “function,” and “emotions” [24]. Symptomatology is considered mild at 8–20 points, moderate at 21–50 points, and severe at 51 points or above [25].

### Statistical analysis

Parametric variables were analyzed using Student’s paired t-test, while non-parametric data were assessed via the Wilcoxon rank-sum test. Linear associations were examined using Spearman’s rank correlation, further validated by linear regression. Significance was set at *p* < 0.05. All analyses were conducted using R-Studio software (version 4.2.2.) [26].

## Results

### Patient characteristics

A total of 69 patients from the DUPIPOLYP (*n* = 43) and IMMUNOPOLYP (*n* = 26) studies were included in the analyses. The patient distribution comprised 23 females (33%) and 46 males (67%). The mean age at the initiation of therapy was calculated as 47.85 years (standard deviation (SD) +/- 12.97), ranging from the youngest patient being 24 years to the oldest 77 years. Seven patients terminated the study prematurely. The reasons for study discontinuation were as follows: unclear reasons (*n* = 3), relocation abroad (*n* = 1), worsening of symptoms (*n* = 1), intensified tinnitus (*n* = 1), and stress (*n* = 1).

Among all patients, 43 completed the SNOT-20 questionnaire, while the SNOT-22 questionnaire was completed by 26 patients. A baseline SNOT score was available for 54 out of the total 69 patients, and baseline NPS data were collected for the entire patient cohort.

### Effects of dupilumab therapy on SNOT scores and NPS

The mean baseline SNOT score at study initiation was 43.3 points, with a SD of 16.8 points. One month after starting dupilumab therapy, the SNOT-20 score exhibited a significant improvement of 19 points (46.57%) (*p* < 0.001, CI 14.5–25.6), while the SNOT-22 score exhibited a decrease of 24.1 points (49.8%) (*p* < 0.001, CI 13.6–37.1) after the first month. Over the course of 3 and 6 months, the SNOT-20 score demonstrated reductions of 19.9 and 22.7 points, respectively, while the SNOT-22 score displayed corresponding declines of 33.8 and 35.7 points. Importantly, in 30 out of 33 patients (90.9%), the SNOT score exhibited a reduction after 3 months relative to the baseline.

**Fig. 1:**
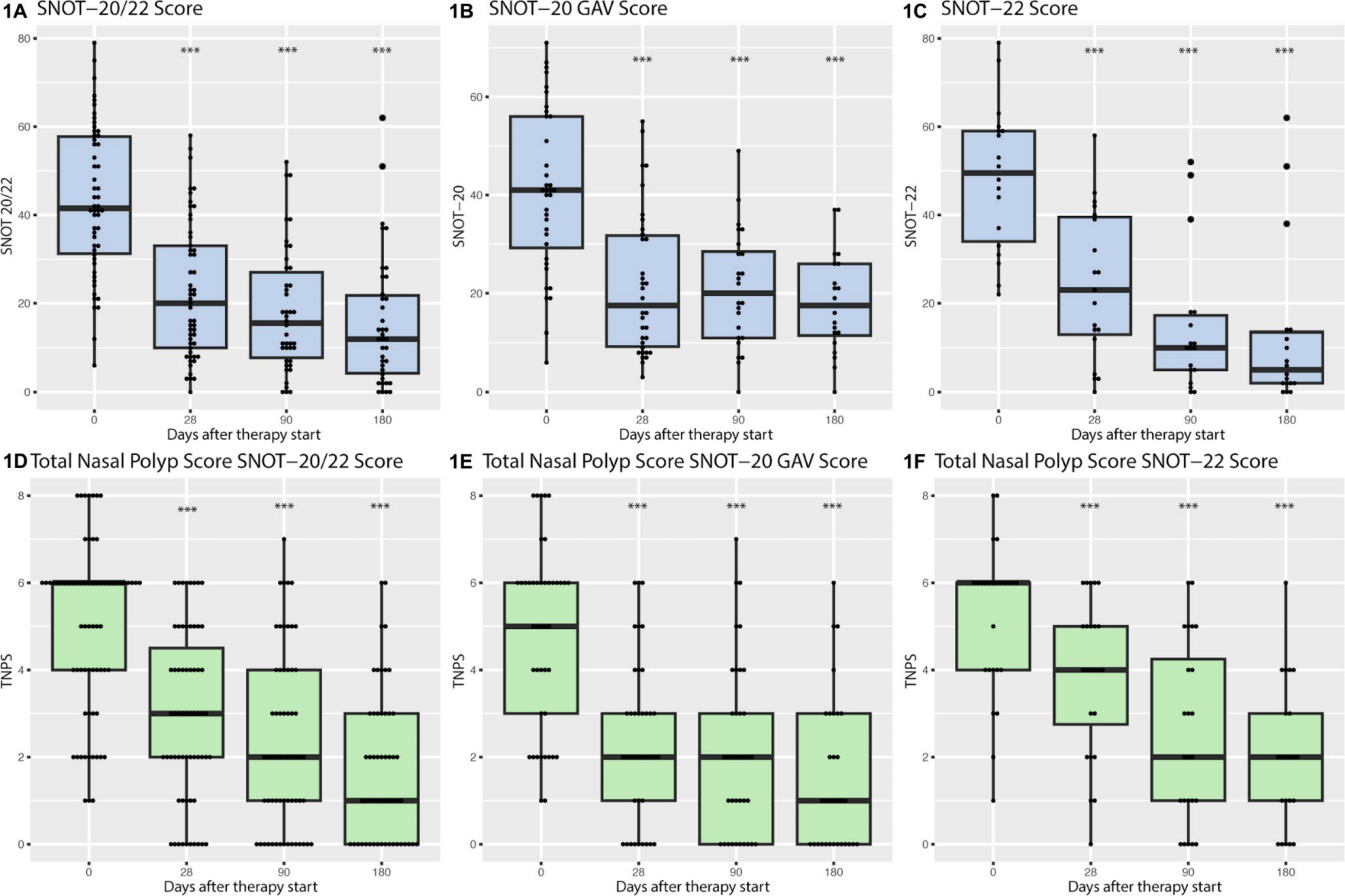
SNOT-20 GAV and SNOT-22 scores with the collected NPS under dupilumab therapy. SNOT-20 GAV or SNOT-22 score (blue) and NPS (green) at days 0, 28, 90, and 180 after initiation of therapy with dupilumab. The whole cohort together (left), DUPIPOLYP cohort (*n* = 43) (middle), IMMUNOPOLYP cohort (*n* = 26) (right). Significant differences in the means or medians of the boxplots from the day 0 reference group are indicated as follows: *p* < 0.05*, *p* < 0.01**, *p* < 0.001***. SNOT: Sino-Nasal Outcome Test, GAV: German Adapted Version, NPS: Nasal Polyp Score. **(A)** SNOT score at days 0, 28, 90, and 180 of the whole cohort (*n* = 69). **(B)** SNOT-20 GAV score at days 0, 28, 90, and 180 (*n* = 43). **(C)** SNOT-22 score at days 0, 28, 90, and 180 (*n* = 26). **(D)** NPS at days 0, 28, 90, and 180 of the whole cohort (*n* = 69). **(E)** NPS at days 0, 28, 90, and 180 of the DUPIPOLYP cohort (*n* = 43). **(F)** NPS at days 0, 28, 90, and 180 of the IMMUNOPOLYP cohort (*n* = 26)

At baseline, the NPS displayed a mean value of 4.97 (median: 6, min: 1, max: 8). Following one month of therapy, a substantial reduction of 3 points (50%) was observed in the NPS (*p* < 0.001, CI 2-2.5). The NPS demonstrated further declines of 2.03 and 2.69 points over the subsequent 3 and 6 months, respectively. Notably, in 50 out of 54 patients (92.6%), an improvement in the NPS was evident after 3 months. In conclusion, a significant trend of improving objective and subjective scores was evident during dupilumab therapy. The results are displayed in Table 1; Fig. 1.

**Table 1:** SD: standard deviation, CI: 95% confidence interval, NPS: nasal polyp score, SNOT: sino-nasal outcome test. The Student’s paired t-test was utilized for SNOT, with SNOT 0 as the reference group. The wilcoxon signed-rank test was applied for NPS, using NPS 0 as the reference group

	Overall (*n* = 69)	SNOT-20 (*n* = 43)	SNOT-22 (*n* = 26)	t-test/W-test *p*-value CI
**SNOT day 0**				
Mean (SD)	43.3 (16.8)	40.8 (16.6)	48.4 (16.6)	
Missing	15 (21.7%)	7 (16.3%)	8 (30.8%)	
**SNOT day 28**				
Mean (SD)	22.7 (15.4)	21.8 (14.6)	24.3 (16.9)	t = 8.48
Missing	16 (23.2%)	9 (20.9%)	7 (26.9%)	*p* < 0.001
				CI (16.7–27.1)
**SNOT day 90**				
Mean (SD)	18.2 (14.0)	20.9 (11.9)	14.6 (15.9)	t = 8.0
Missing	27 (39.1%)	19 (44.2%)	8 (30.8%)	*p* < 0.001
				CI (20–33.7)
**SNOT day 180**				
Mean (SD)	15.6 (14.7)	18.1 (10.2)	12.7 (18.4)	t = 7.85
Missing	31 (44.9%)	23 (53.5%)	8 (30.8%)	*p* < 0.001
				CI (21.2–36)
**TNPS day 0**				
Median (Min, Max)	6 (1, 8)	5 (1, 8)	6 (1, 8)	
Missing	0 (0%)	0 (0%)	0 (0%)	
**TNPS day 28**				
Median (Min, Max)	3 (0, 6)	2 (0, 6)	4 (0, 6)	W = 1418
Missing	6 (8.7%)	4 (9.3%)	2 (7.7%)	*p* < 0.001
				CI (2–2.5)
**TNPS day 90**				
Median (Min, Max)	2 (0, 7)	2 (0, 7)	2 (0, 6)	W = 1633
Missing	8 (11.6%)	6 (14.0%)	2 (7.7%)	*p* < 0.001
				CI (2.5–3.5)
**TNPS day 180**				
Median (Min, Max)	1 (0, 6)	1 (0, 6)	2 (0, 6)	W = 1275
Missing	15 (21.7%)	11 (25.6%)	4 (15.4%)	*p* < 0.001
				CI (3–4)

### Correlation of the absolute values of the SNOT score with the NPS

**Fig. 2:**
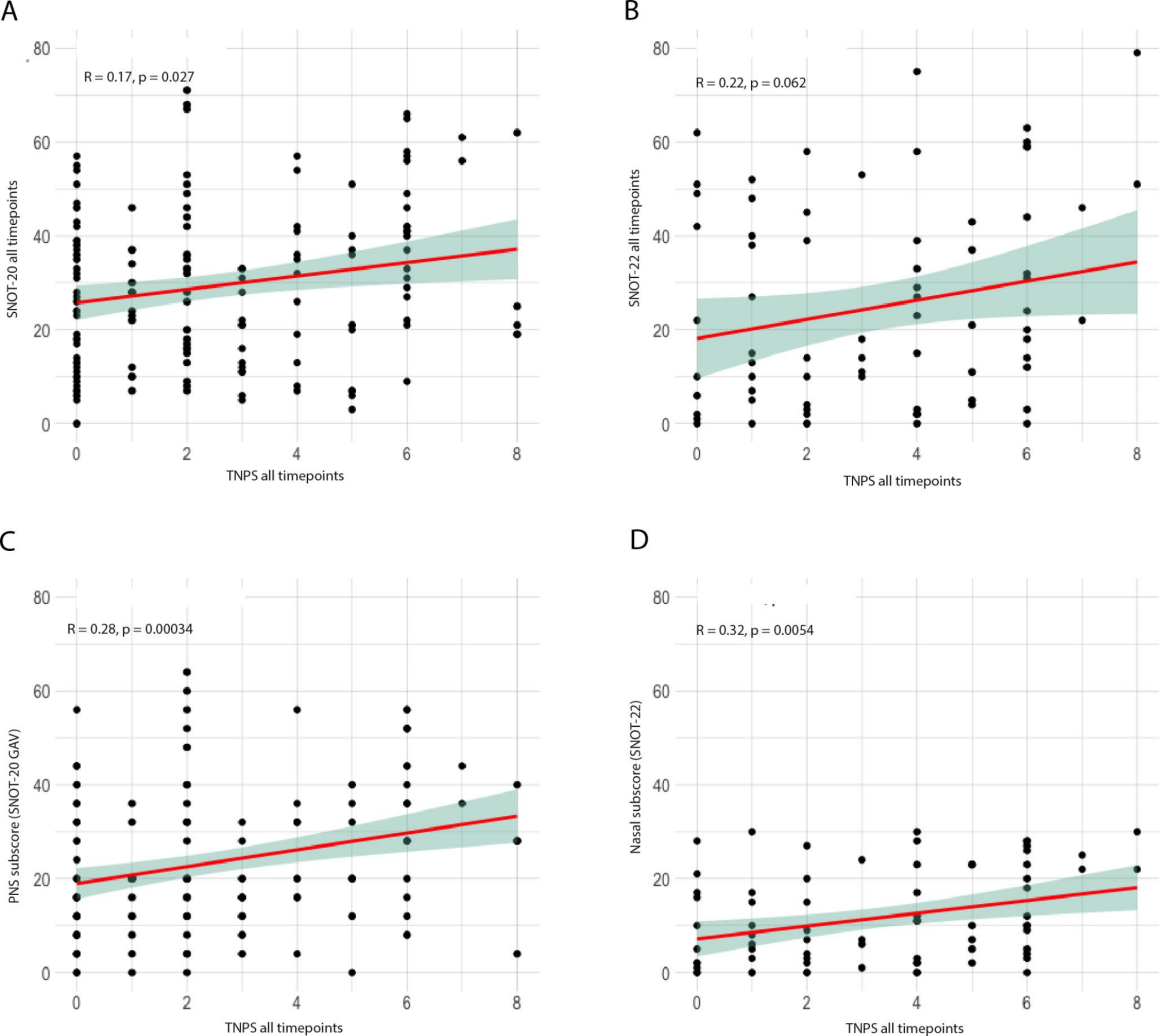
Correlation of the absolute values of the SNOT-20 GAV or SNOT-22 score to the NPS independent of the time of assessment. Spearman’s correlation of absolute values between SNOT-20 GAV **(A)** and SNOT-22 **(B)** and NPS regardless of the time of assessment. Spearman’s correlation of the absolute values of the SNOT-20 GAV PNS subscore **(C)**, SNOT-22 nasal subscore **(D)**, and the NPS at all time points. The linear trend is plotted as a red line with the 95% confidence interval as a green shadow. Spearman’s Rho as well as its significance are shown in the upper left of each. SNOT: Sino-Nasal Outcome Test, GAV: German Adapted Version, NPS: Nasal Polyp Score, PNS: Primary Nasal Symptoms. R = Spearman’s Rho, *p* = *p*-value

Correlating the absolute SNOT and NPS scores, regardless of the time point and therapy course, only the SNOT-20 score exhibited a linear, very weak trend (*r* = 0.17, *p* = 0.027). The SNOT-22 score did not show a significant correlation with the NPS, but fewer patient data were captured for this analysis (SNOT-20: 169 data points, SNOT-22: 77 data points) (Fig. 2a-b).

When analyzing the correlations between the SNOT subscores and NPS, significant correlations were observed exclusively for the PNS subscore in the SNOT-20 score (*r* = 0.28, *p* < 0.001) and the nasal subscore in the SNOT-22 score (*r* = 0.32, *p* = 0.005) (Fig. 2c and d).

### Correlation of the absolute changes over time of the SNOT score with the NPS

Analyzing the absolute change over time did not yield significant results: Difference from Day 0 to Day 90 NPS vs. SNOT-20 (*r* = 0.14, *p* = 0.58), NPS vs. SNOT-22 (*r* = 0.065, *p* = 0.81), difference from Day 0 to Day 180 NPS vs. SNOT-20 (*r* = 0.0098, *p* = 0.97), NPS vs. SNOT-22 (*r* = 0.05, *p* = 0.86) (data not shown). In summary, the absolute change in NPS did not correlate with the absolute change in SNOT scores.

### Correlation between the relative changes in SNOT score and NPS, irrespective of the timing

**Fig. 3:**
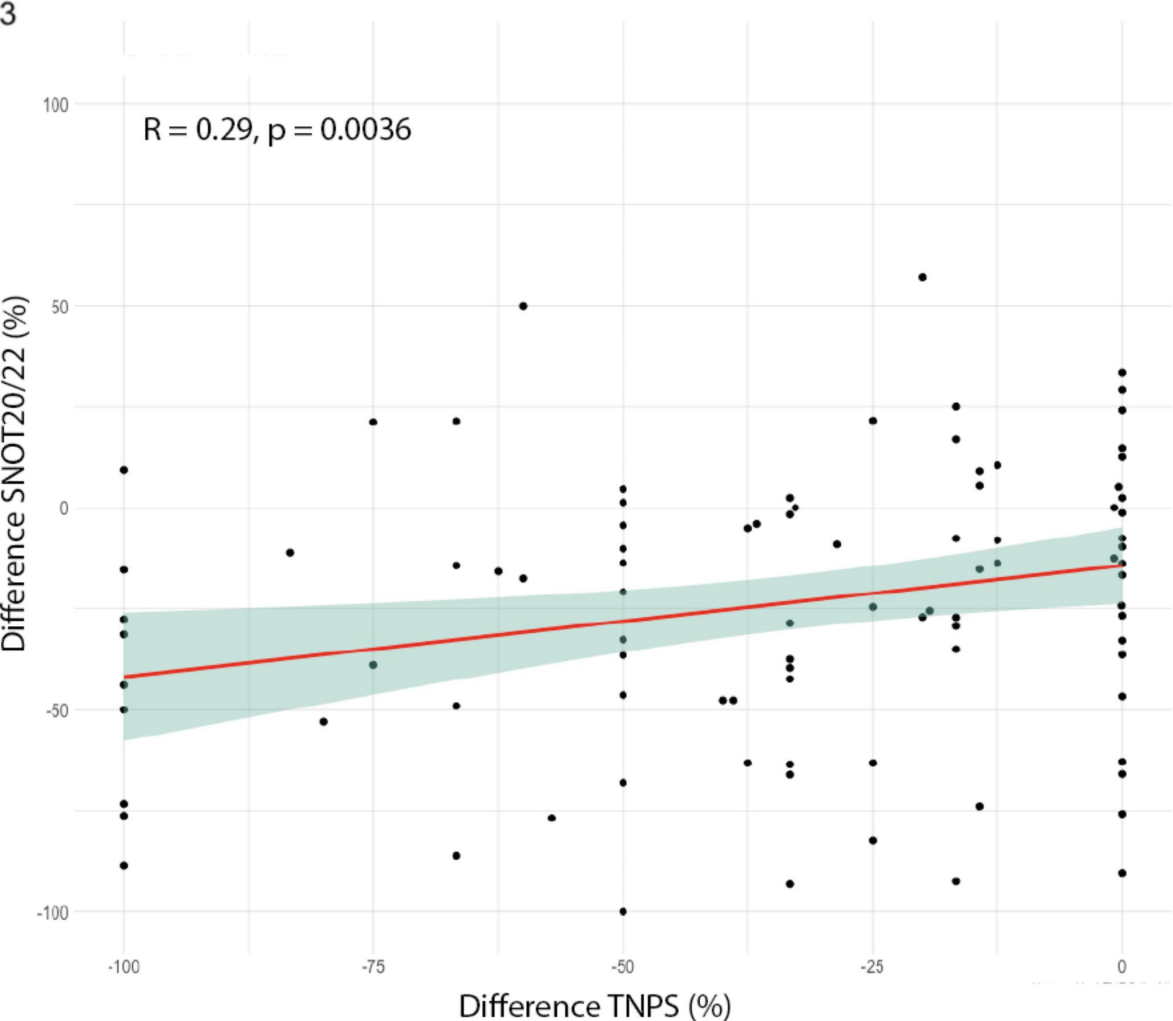
Correlation of percentage values of SNOT-20/22 score to NPS independent of time point. Relative decrease/increase in SNOT score 20/22 (y-axis) correlated (Spearman) with the relative decrease in nasal polyp score (x-axis). Differences were calculated from days 0–28, 29–90, and 91–180, for each patient. Consequently, each patient contributed three data points to the analysis. The red line shows the linear trend with the 95% confidence interval as shading. Spearman’s Rho and its significance are shown in the top left corner of each case. SNOT: Sino-Nasal Outcome Test, NPS: Nasal Polyp Score. R = Spearman’s Rho, p = p-value

To assess the SNOT score independently of its baseline value, the relative differences between time points Day 0–27, 28–90, and 91–180 were computed and compared with the relative changes in the polyp score during these intervals, enabling a joint evaluation of the SNOT-20 and SNOT-22 scores. A significant positive correlation was identified (*r* = 0.29, *p* = 0.004). The analysis indicated that a complete reduction of nasal polyps by 100% corresponds to a statistically significant decrease of 32% in the SNOT score. A 50% decrease in polyps, was associated with a reduction of 25% in the SNOT score. Furthermore, even in cases where the NPS did not improve, there was an evident 18% improvement in the SNOT score. However, employing a linear regression model did not yield conclusive evidence for the influence of NPS reduction on SNOT score changes. The calculated regression coefficient, with a potential for predicting SNOT score improvement, is 0.141 (*p* = 0.12, SE = 0.09, y = 0.141*x+(-18.07)). In summary, they exhibit correlation but lack predictive capability. The findings are visually represented in Fig. 3.

**Fig. 4:**
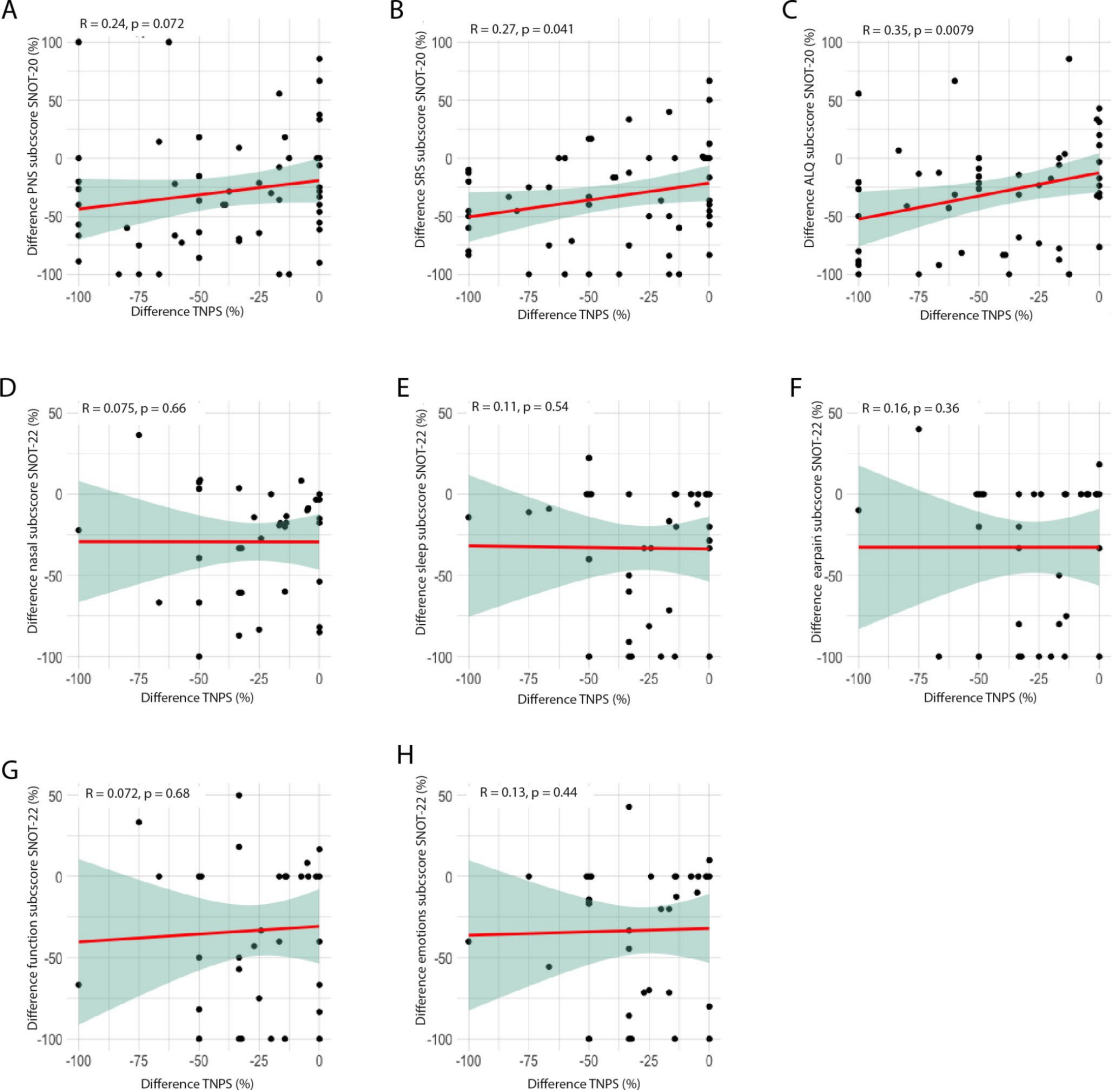
Percentage decrease/increase in SNOT-20 GAV and SNOT-22 subscores correlated (Spearman) with percentage decrease in NPS. **(A)** SNOT-20 subscore “primary nasal symptoms”. **(B)** SNOT-20 subscore “secondary rhinogenic symptoms”. **(C)** SNOT-20 subscore “general quality of life”. **(D)** SNOT-22 subscore “nasal symptoms”. **(E)** SNOT-22 subscore “sleep”. **(F)** SNOT-22 subscore “earache”. **(G)** SNOT-22 subscore “function”. **(H)** SNOT-22 subscore “emotions”. Differences were calculated from day 0–28, 28–90, and 90–180. The red line shows the linear trend with the 95% confidence interval as shading. Spearman’s Rho as well as its significance are shown in the upper left corner of each case. SNOT: Sino-Nasal Outcome Test, GAV: German Adapted Version, NPS: Nasal Polyp Score. R = Spearman’s Rho, *p* = *p*-value

When analyzing the SNOT score using the same method described above but subdivided into its subscores, we observed that the PNS subscore displayed no correlation, as illustrated in Fig. 4a. Nonetheless, a marginal, but statistically significant improvement in the SRS and ALQ subscores was noted concurrently with a reduction in nasal polyps, as depicted in Fig. 4b and c. Conversely, when examining the SNOT-22 subscores, our investigation revealed an absence of correlation between the percentage difference in subscores and the relative reduction of the NPS (Fig. 4d-h).

### Correlation between the relative changes in SNOT and NPS, stratified by the baseline NPS

Since both the baseline SNOT score and the baseline NPS exhibit significant variability, patients were grouped based on the polyp grade at day 0, (NPS 8: 7 patients, NPS 6: 26 patients, NPS 4: 10 patients, NPS 2: 9 patients). Participants with NPS 7 (*n* = 4), 5 (*n* = 6), 3 (*n* = 4) und 1 (*n* = 3) were excluded from the analysis due to insufficient sample size.

**Fig. 5:**
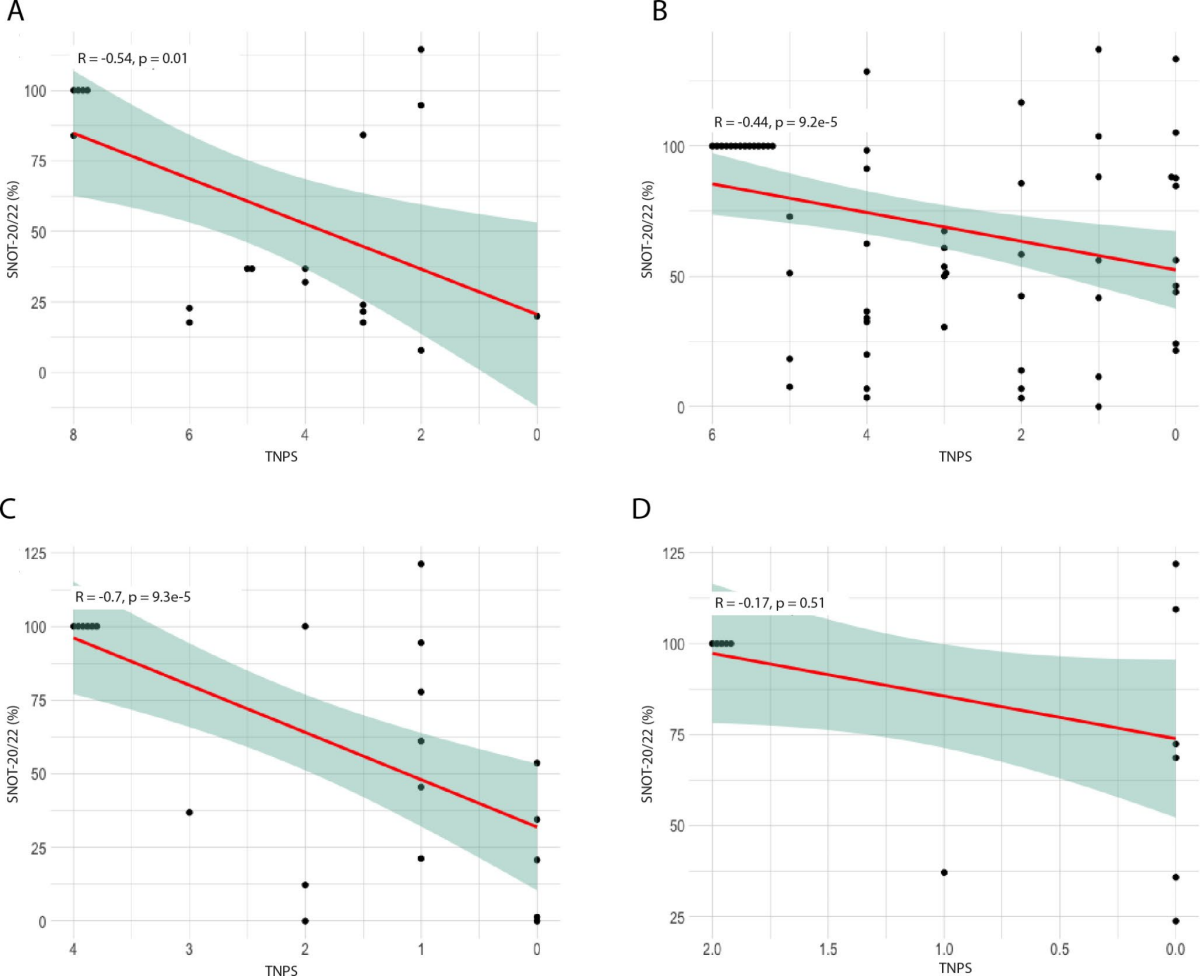
Correlation of percentage of SNOT-20/22 score to NPS divided by baseline NPS at start of therapy. Patients were classified according to their NPS at baseline. The baseline SNOT score for each patient was set at 100%, irrespective of its absolute value. Then, the decrease/increase in SNOT-20/22 score was correlated to the percent decrease in nasal polyp score. The red line shows the linear trend with the 95% confidence interval as a shadow. SNOT: Sino-Nasal Outcome Test, NPS: Nasal Polyp Score. R = Spearman’s Rho, *p* = *p*-value. **(A)** All Patients with NPS 8 at baseline (*n* = 7). **(B)** All Patients with NPS 6 at baseline (*n* = 26). **(C)** All Patients with NPS 4 at baseline (*n* = 10). **(D)** All Patients with NPS 2 at baseline (*n* = 9)

The baseline SNOT score for each patient was set at 100% irrespective of its absolute value. Subsequently, the correlation between the changes in SNOT and NPS was examined. In all groups, a significant correlation between the relative change in SNOT score and the NPS was observed. The correlation was: *r* = -0.54 (*p* = 0.01), *r* = -0.44 (*p* < 0.001), and *r* = -0.7 (*p* < 0.001) for the groups with baseline NPS of 8, 6, and 4, respectively. However, no significant association between the two scores was found in the group with an NPS of 2 at the initiation of therapy (*r* = -0.17, *p* = 0.51). The results are displayed in Fig. 5a-d. The correlation was further validated through a linear regression model: NPS 8: y = 8.03x + 20.57 SE 2.75, *p* = 0.008, NPS 6: y = 5.5x + 52.42, SE 1.77, *p* = 0.003, NPS 4: y = 16.05x + 31.91, SE 3.8, *p* < 0.001. In summary, stratifying the scores by baseline NPS reveals correlations and even yields evidence of predictive capability.

## Discussion

### Main results

Patients responded rapidly to dupilumab therapy. Correlation analysis of absolute values revealed no significance. Specifically, a decrease in absolute NPS doesn’t necessarily correspond to a simultaneous reduction in the absolute SNOT-22 score. Similarly, the observed weak trend between NPS and SNOT-20 underscores its negligible relevance. Furthermore, analyzing absolute changes over time yielded no correlations. Our method for evaluating relative changes involves considering the percentage shift in scores in relation to the baseline. Relative changes in NPS and SNOT scores are statistically connected, yet regression analysis indicated non-significant predictive value. However, stratifying patients by baseline NPS revealed a significant correlation as well as predictive capability between NPS and relative SNOT score change in most groups (NPS 4, 6, 8).

### Effect of dupilumab therapy on scores

Our findings align with existing evidence supporting the positive impact of dupilumab on SNOT and NPS scores [27, 28]. In our study, a significant average decrease of 24.1 points in the SNOT-22 score was observed after just one month. This reduction surpasses the minimal clinically important difference (MCID) reported by Hopkins et al. (-8.9 points) [29], and even exceeds the MCID of -12 points for medically managed CRS patients established by Phillips et al. [30]. Consequently, it can be concluded that Dupilumab not only demonstrated statistically significant enhancement but also yielded clinically meaningful improvement (as measured by PROMS).

### Correlation analyses of absolute values

Even though objective scores and PROMs tend to decrease drastically under dupilumab therapy, the correlation between them has not been comprehensively explored in previous studies. Hence, Ta et al. 2021 conducted a systematic review examining the relationship between objective outcome measures and PROMs [15]. Nasal endoscopic ratings failed to exhibit any statistically significant correlations with PROMs. Consistent with these findings, other studies also arrived at a similar conclusion, indicating a lack of correlation between widely used endoscopic scoring systems and SNOT-22 scores [31, 32]. Aligning with these results, our analysis of absolute score values revealed no significant correlation between SNOT-22 score and NPS.

Recent research specifically focusing on dupilumab therapy in CRSwNP patients has demonstrated that NPS scoring correlates with objective measures, such as the SST-12. However, no significant correlations were observed between the subjective SNOT scores and olfactory function as assessed by the SST-12 [33].

Examining SNOT-20, we identified a subtle correlation trend between SNOT-20 and NPS (*r* = 0.17, *p* = 0.027). This observation aligns with analogous findings reported by a separate study, which also documented a weak trend (*r* = 0.33, *p* = 0.02) [34]. Conversely, other authors reached a divergent conclusion, that the correlation coefficient was nearly zero, suggesting a random relationship between SNOT-20 and endoscopy findings [35].

Focusing on subscores, our analysis unveiled significant correlations exclusively for the PNS subscore in SNOT-20 and the nasal subscore in the SNOT-22. These findings are consistent with previous literature, highlighting stronger correlations between polyp scores and nasal domains within the SNOT [36–38]. It’s suggested that the historically observed low correlations between endoscopic findings and PROMs may stem from the use of aggregate scores, thereby diluting meaningful correlations [37]. Thus, as might be intuitively expected, the NPS exhibits better predictive capacity for symptoms, particularly within nasal domains, when analyzed separately.

### Correlation analyses of absolute changes over time

Jeong et al. conducted a 2022 meta-analysis, examining all previously used endoscopic nasal polyp scoring systems to assess their correlations with PROMs [19]. They employed the approach of correlating absolute changes over time, but still found no statistical significance. Our analysis yielded similar results, as statistically significant evidence was absent when correlating the absolute change in NPS with the absolute change in SNOT scores.

While a rapid initial response under dupilumab might suggest a monotonic trend, such a trend should still be detectable by Spearman’s rank correlation, which is sensitive to both linear and monotonic associations. Therefore, the lack of correlation in our study cannot be attributed to the quick response. It likely reflects variability in subjective symptom perception, which can obscure the relationship between NPS and SNOT scores.

### Correlation analyses of relative changes

To the best of our knowledge, no other study has employed an approach similar to evaluating relative changes rather than absolute changes. Correlating the relative changes of PROMs with nasal polyp severity revealed significant results. Moreover, stratifying patients by baseline NPS revealed a significant correlation between NPS and relative SNOT score change in most groups (NPS 4, 6, 8) except for NPS 2 at baseline. These divergent results, whether considering absolute or relative values, can be explained by the fact that patients’ subjective perception of disease may differ greatly and patients with the same baseline NPS have a wide range of different baseline SNOT scores. Examining the relative differences in scores, rather than the absolute ones, reveals a significant percentage improvement in SNOT score with a concomitant percentage decrease in polyp score with dupilumab therapy, which represents a novel and clinically relevant finding. Opposing the conclusion that new nasal polyp grading systems with improved clinical utility are necessary because of their limited predictive capability [19], we argue that the lack of correlation between PROMs and NPS cannot be attributed to their limited reliability. Instead, we reemphasize that the subjective perception remains highly variable even at the same disease burden, making correlation analyses with relative changes stratified by baseline scores more appropriate. Introducing a new scoring system merely contributes to an expansion of scoring methods, potentially complicating cross-study comparisons when different measures are employed across various studies.

At this point, we also want to emphasize that it is unwarranted for insurance companies to consistently require absolute SNOT and polyp scores.

### Strengths and limitations

A notable strength of our study is that it represents the first comprehensive attempt to employ relative and percentage measures in analyzing the correlations between objective scores and PROMs. However, some limitations should be considered. Participants were required to complete the SNOT questionnaire weekly, but inconsistent compliance may have impacted symptom assessment accuracy. The two questionnaires are essentially identical. The difference is that the SNOT-22 contains two additional questions: “Lack of good night’s sleep” and “Wake up tired”. In addition, one question differs between the two questionnaires: “Need to throat clearing” in the Snot-20 GAV and “Need to blow nose” in the SNOT-22.

Additionally, interpreting rhinoscopy findings for nasal polyp scoring can be challenging under certain conditions, such as severe mucosal swelling or limited patient cooperation. Changes in polyp size during therapy may lead to paradoxical shifts in polyp scores, i.e. revealing polyps between the middle turbinate and septum, allowing for potential bias. Furthermore, although we reviewed the videos in cases of uncertainty, a residual risk of inter- and intra-observer variability remains, as the assessment is inherently subject to the subjective judgment of the examiners. Previous surgery may also interfere with the NPS grading system, potentially introducing further biases.

## Conclusion

Dupilumab therapy demonstrated substantial improvements in SNOT and NPS. However, our findings highlight the limitations of absolute correlation analyses, revealing only a weak trend for SNOT-20 and correlations only within the subscore analysis of the nasal subscores. The limitations of absolute correlation analysis are likely influenced by the inherent variability in subjective perception. Hence, it appears more suitable to correlate relative changes and to stratify patients based on their baseline values. Therefore, to ultimately enhance our understanding of CRSwNP treatment outcomes, future research should continue to explore the utility of relative change correlation analyses.

## Declarations

### Oxford centre for evidence-based medicine 2011

Level 3 of Evidence.
